# The importance of an ecologically valid method in the evaluation of toddler interaction with coloured liquid laundry detergent capsules

**DOI:** 10.1371/journal.pone.0199976

**Published:** 2018-07-02

**Authors:** Annalise Richmond, Zhiwu Liang, Valmire Mulaj, Jan Ryckmans, Gerard Stijntjes

**Affiliations:** 1 Procter and Gamble, Strombeek Bever, Belgium; 2 haystack NV, Heverlee, Belgium; University of Melbourne, AUSTRALIA

## Abstract

The colour and appearance of liquid laundry capsules have been implicated in the risk of attracting the attention of toddlers, and therefore contributing to poisoning incidents in the home by encouraging interaction. This research set out to explore if differences in colours and contrasting colour designs used in mono and multi-coloured capsules result in different levels of attractiveness. This was performed using two study settings: a laboratory setting (out of context), and by comparison, a more ecologically valid setting, mimicking the real-world. Capsule attractiveness to toddlers was measured by visual attention (measured through eye tracking) and grasping choice (measured as frequency of grasping in a behavioural task). Results from the research in the out of context setting showed statistically significant differences in visual attention and grasping choice between colours and contrasting designs. In the visual attention study a preference for multi-coloured capsules was shown. In the grasping choice study, in addition to multi-coloured, mono-coloured white or purple capsules were also preferred. In the more ecologically valid setting, there were no statistically significant differences in the visual attention or grasping choice between any of the capsules. These results were consistent with each other and in line with market data reflecting poisoning incident rates, which show no change with colour or contrasting colour design. We suggest that the results from out of context studies might not be a reliable indicator of real world behaviour. Given the importance of toddler home safety, using a methodology that is aligned with market numbers is crucial to develop countermeasures.

## Introduction

Media news reports have highlighted safety concerns with colours used in liquid laundry capsules, implying that these are attractive to toddlers and hence may contribute to unintentional interaction that could lead to poisoning incidents in the home [1, 2]. On the other hand, reported poisoning incidents have been associated with mono-colour capsules since they were first launched in 2001 [3, 4] and levels (adjusted to exposure baseline taking into account volume of sales) have not changed with differing colours or the introduction of multi-coloured designs.

Previous studies on the early development of colour preference in children are inconsistent and only limited data are available that explain colour preference in toddlers [5, 6]. Older studies have relied on trained observers to determine the length of time toddlers spent looking at stimuli [7]; whilst others have relied on toddlers’ natural tendency to pick up objects that catch their attention [8]. More recent studies have used eye tracking equipment to quantify eye movements and length of time spent looking at stimuli [9]. This technological advance has allowed for the measurement of toddler looking behaviour to be recorded automatically and more precisely than has been possible before, providing investigators with a powerful tool for measuring not only how long toddlers look at stimuli, but precisely where they look and how they visually scan those stimuli [10]. This capability now enables the tracking of visual attention in more ecologically valid experimental environments.

This research set out to formally explore if differences in colours and contrasting colour designs used in mono and multi-coloured capsules result in different levels of attractiveness to toddlers and to explore the impact of a more ecologically valid study design. Using a controlled lab set-up in combination with a more ecologically valid approach will allow us to explore whether previously observed effects occur under typical conditions for the population at large rather than being limited to a laboratory set-up [11]. The current research measures both visual attention and behavioural interaction (i.e. grasping) with the ultimate goal to understand accidentology data, and tests whether both out of context and a more ecologically valid design yields the same result. The focus of the study was on toddlers aged 12–36 months, as this is the age group at greatest risk of poisoning incidents based on industry incident reports [4, 12, 13].

## Materials, methods and results

**Fig 1:**
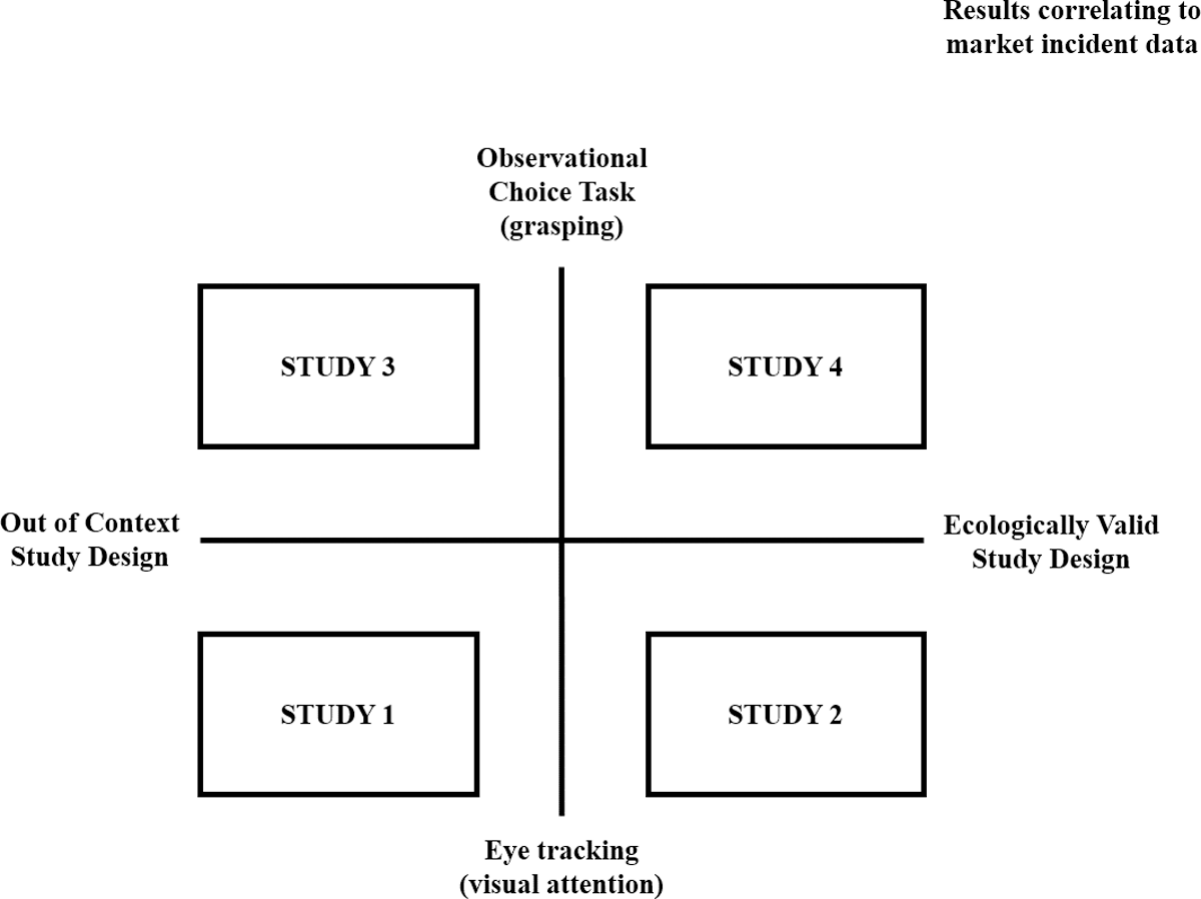
Overview of study designs.

The research was comprised of four studies–study 1 to 4. Studies 1 and 2 were carried out in the first week of field work and studies 3 and 4 the second following week. Participants taking part in study 1 and 2 also took part in study 3 and 4 together with additional toddlers, required to increase the statistical robustness of studies 3 and 4 whose design divided the toddlers into two independent groups. Participants were recruited from the local community in Frankfurt, Germany representing a cross section of local race and demographics. This study was reviewed and approved in advance by Procter & Gamble’s Independent Human Safety Compliance Department (Brussels Innovation Centre, 1853 Strombeek Bever, Belgium) ref number ELN EXP-16-BI0420, and informed consent was provided by all parents of panellists in writing. In equivalence to university ethics committees, these procedures ensure consistency in approach as well as the protection of the rights, safety, and well-being of all test subjects as well as assuring that P&G’s human testing research program is in compliance with the regulatory requirements, ESOMAR world research codes, Company business and ethical standards, and the Declaration of Helsinki. An overview of the study designs can be found in Fig 1.

**Fig 2:**
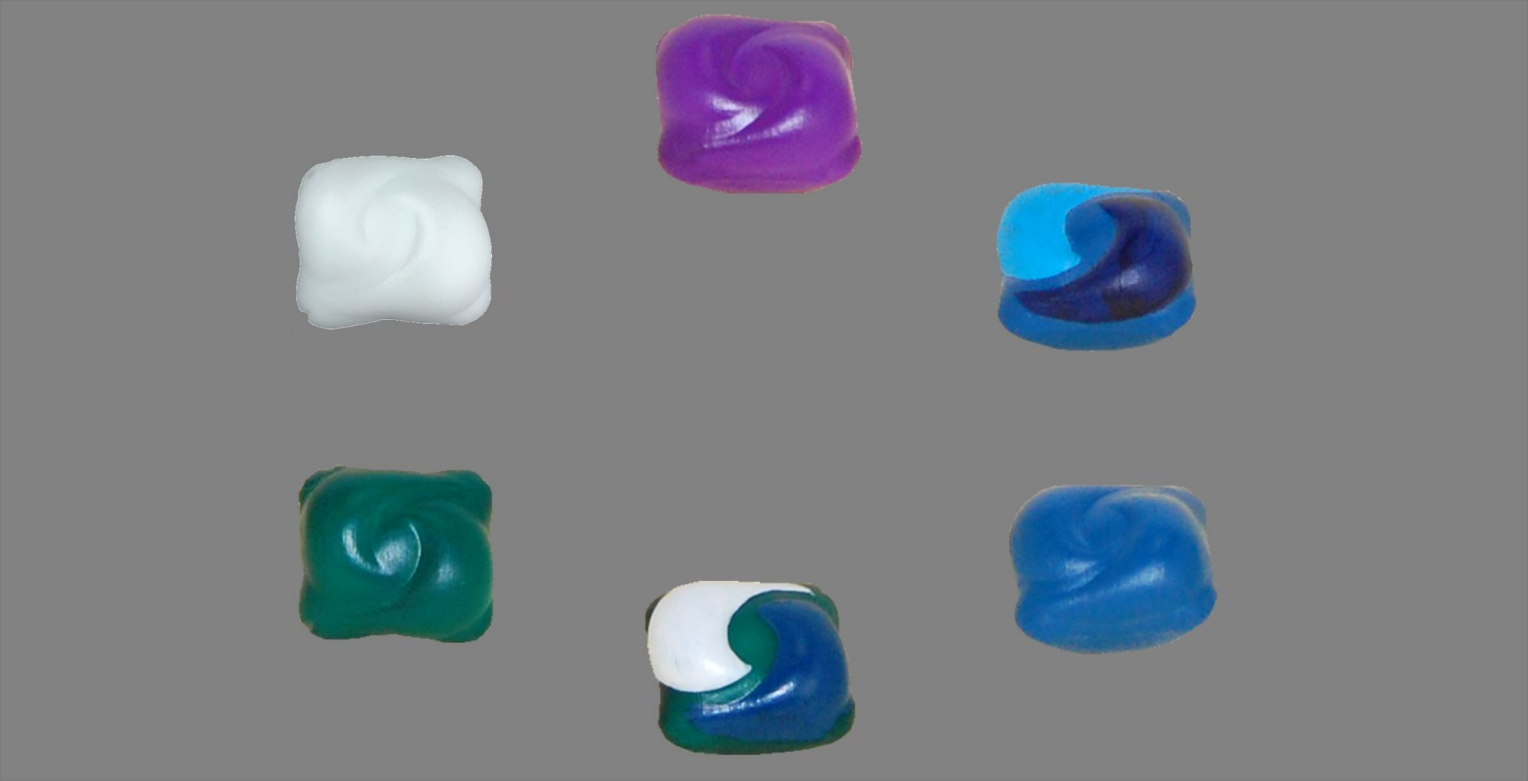
Image of liquid laundry capsules arranged in a circle (study 1).

Study 1: Out of context eye tracking study–examining differences in toddlers’ visual attention to a high-resolution image of different coloured liquid laundry capsules when set in a circle alongside one another on a neutral background (Fig 2)

**Fig 3:**
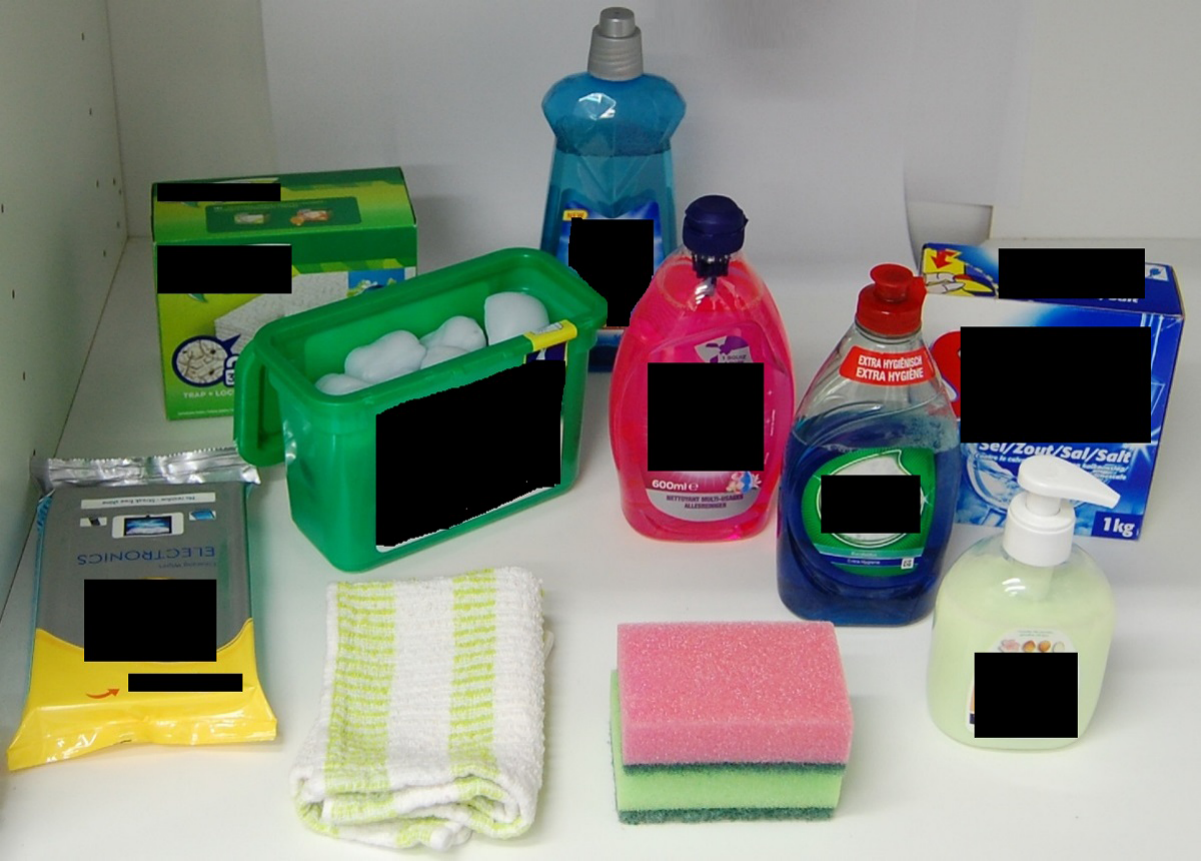
Image of kitchen cupboard set-up–white liquid laundry capsules (study 2).

**Fig 4:**
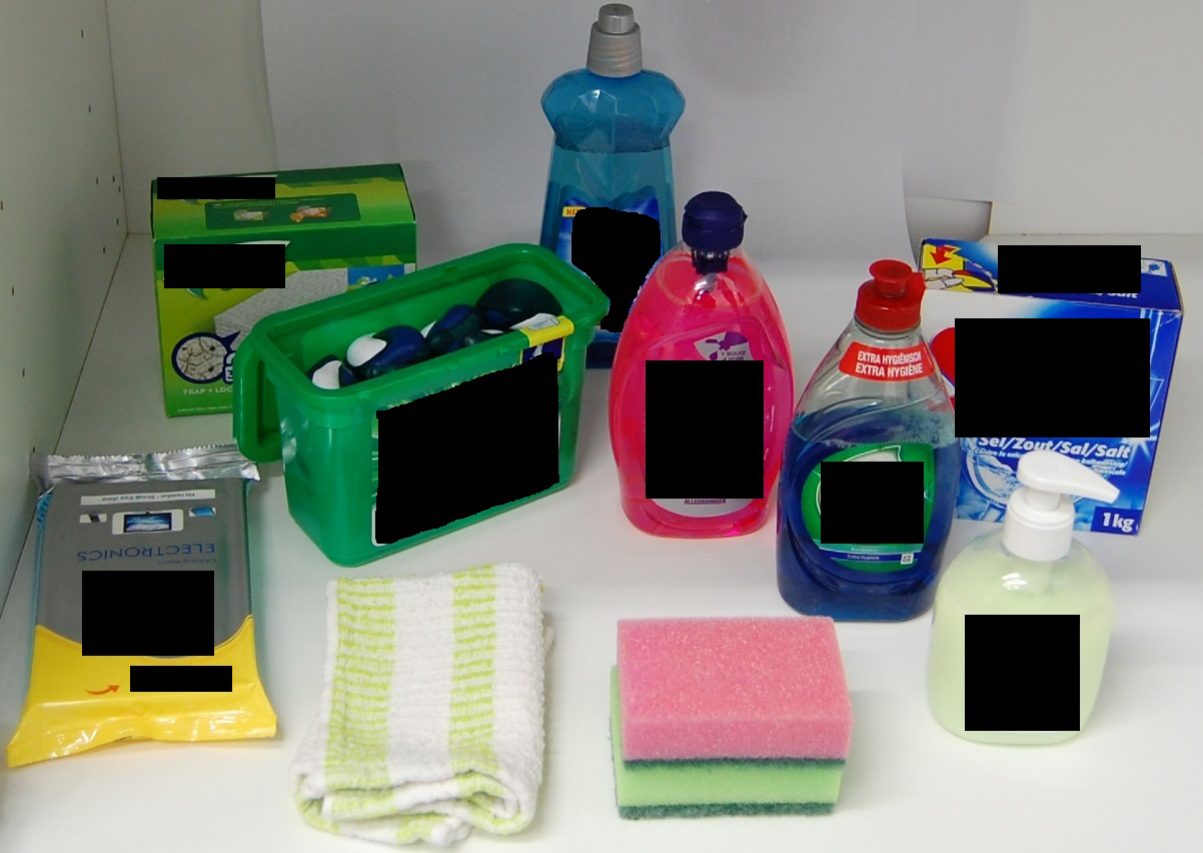
Image of kitchen cupboard set-up–high contrast liquid laundry capsules (study 2).

Study 2: A more ecologically valid eye tracking study–examining differences in toddlers’ visual attention to a high-resolution image of different coloured liquid laundry capsules when presented in their open packaging (box) in an “under the sink” kitchen cupboard set-up, alongside other household cleaning items. This was designed to mimic that of typical storage in European households (Figs 3 and 4)

**Fig 5:**
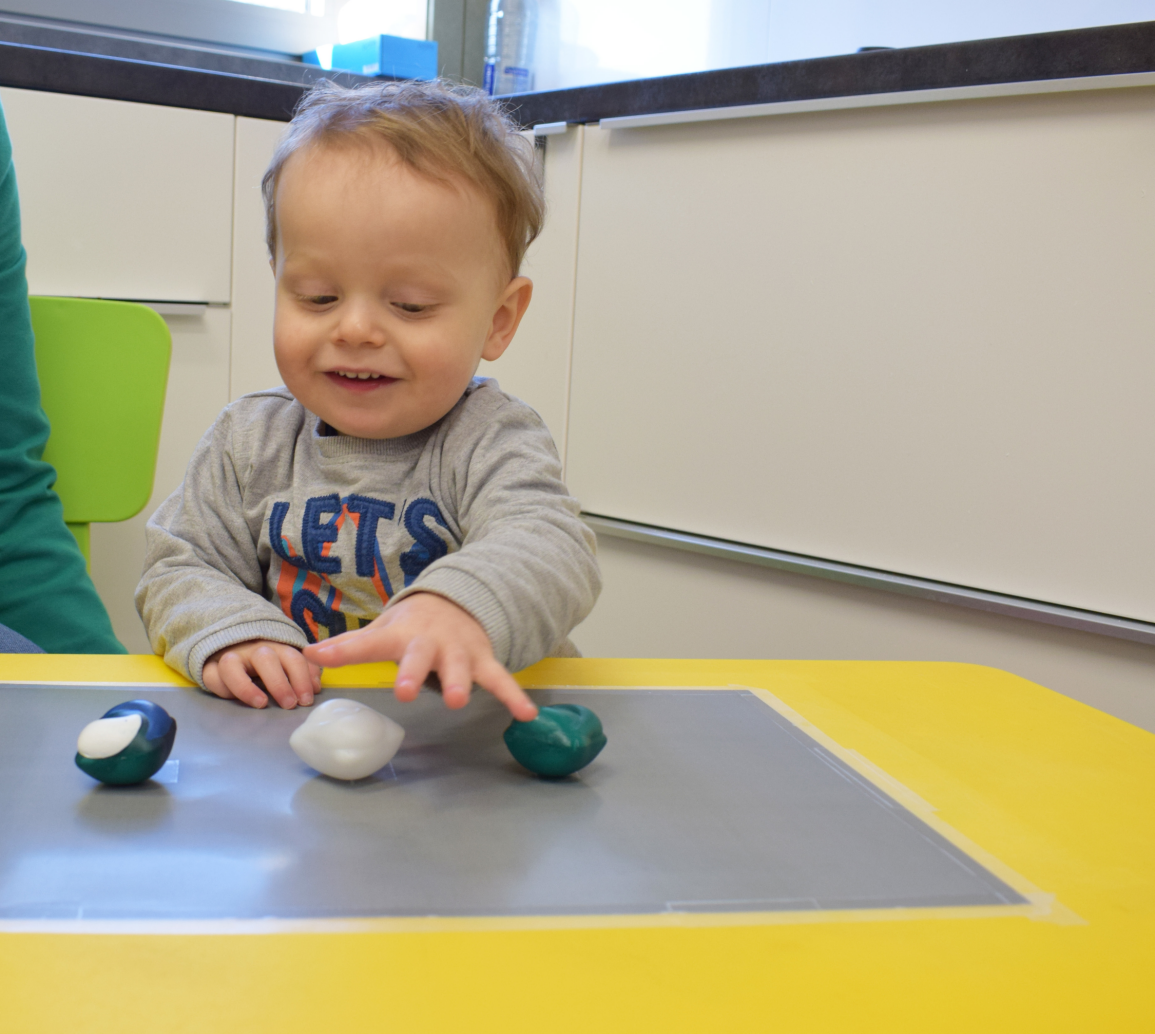
Toddler taking part in out of context behavioural observation task (study 3).

Study 3: Out of context behavioural observation study–examining differences in toddlers’ grasping choice when presented with 3D plastic replicas of differently coloured liquid laundry capsules set in a line alongside one another on a neutral background (Fig 5)

**Fig 6:**
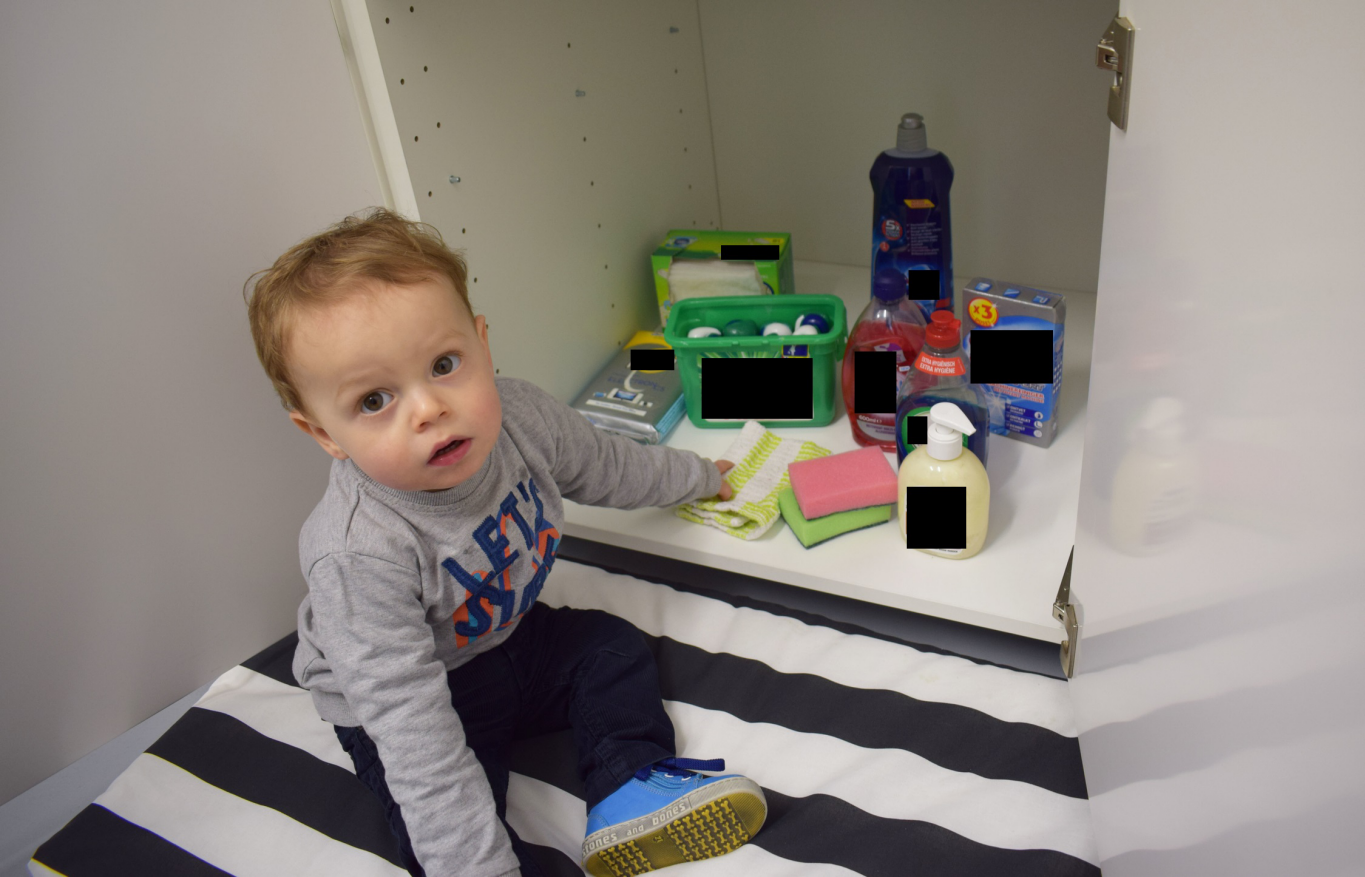
Toddler taking part in a more ecologically valid behavioural observation task (study 4).

Study 4: A more ecologically valid behavioural observation study–examining differences in toddlers’ grasping choice when presented with 3D plastic replicas of differently coloured liquid laundry capsules in their open packaging (box) in an “under the sink” kitchen cupboard set-up alongside other household cleaning items. This was designed to mimic that of typical storage in a European household, as per Study 2 (Fig 6)

### Study 1 –Out of context eye tracking study

Method: On a television monitor, each toddler viewed a single high-resolution image of six different colour capsules arranged in a circle (Fig 2). The image was projected for four seconds. Four seconds was chosen based on an agreed acceptable time to display the images that was sufficient for the toddler to look, yet not too long so that the toddler would be able to look at everything in the image leading to the difference between the times spent looking at the different colours would becoming less distinct. This study measured visual attention using the fixation duration (seconds) and percent noticed rate (%).

Participants: 134 toddlers (mean age in months and standard deviation (M ± SD), 21.59 ± 6.41; 63 boys, 71 girls; range (R), 12–36) were assigned to the task. A total of 23 toddlers were excluded from the study for not meeting the inclusion criteria for sufficient eye tracking data (see below for a more detailed description of these criteria). The data were collected from the remaining 111 toddlers (M ± SD, 21.70 ± 6.46; 53 boys, 58 girls; R, 12–36).

Stimulus materials: Replica plastic 3D printed capsules of equal size, weight and shape were made. These were painted and varnished with one finish to visually represent, as closely as possible, common capsule colours (hue, saturation, brightness). These colours were mono-colour green, blue, white, purple and multi-coloured low and high contrast (light blue/blue/dark blue and green/blue/white). One capsule replica representing each of the six colour/colour combinations were arranged in a circle on a neutral grey background. The ordering and sequence of the capsules in the circle was randomised using SAS9.4 software to ensure that each individual colour was in a different position. High resolution images for each position were taken and a single shot was projected individually to each participant on a television monitor following the randomisation pattern.

Experimental set-up: A remote infrared eye tracker was used (Tobii X120, Tobii Technology, Danderyd, Sweden). This equipment registers the focal gaze (x,y position) every 12 milliseconds (sampling frequency: 120 Hz), and can distinguish spatial changes when the gaze angle differs by 0.3 degrees. The data were used to calculate the direct notice rate of capsules and fixation duration, using Tobii Studio version 3.4.5 software. Looking at a specific capsule (direct noticing) was inferred when the output indicated that the focal gaze of the toddler fell directly on the capsule in the image. Fixation duration was defined as the time spent looking at any given capsule within the 4 second stimulus display. The quality of the recording was checked for every image, and data was excluded for toddlers who did not have any gaze data or who had a large offset (i.e. consistently looking below/above or beside the products). Toddlers with a total fixation duration below 0.75 seconds were excluded from the analyses.

Procedure: All eye tracking sessions were conducted in the presence of the accompanying parent, with the toddler sitting on their parent’s lap during the session to minimise stress and maintain a relatively fixed position in relation to the eye tracker, at eye level and 75–100 cm from the screen of the television monitor. The toddler’s gaze was initially calibrated using a picture of a kitten, shown at five specific points on the screen (a minimum of three points were needed for calibration). Study 2 was completed first and all toddlers went on to immediately complete this study and within the same research conditions.

Statistical Analysis: A mixed model was used for statistical analysis of fixation durations. Within this model the capsule colour, order within the circle and sequence of the six different coloured capsule stimuli were taken as fixed effects, the participant as a random effect and fixation duration as a response variable. Tukey Honest Significant Difference (HSD) was then used for post-hoc analysis pairwise comparisons across the six coloured capsules (i.e. blue vs. green, blue vs. purple, blue vs. white, blue vs. high contrast, blue vs. low contrast, green vs. purple, etc.)–with 15 paired comparisons in total. JMP 13.1 was used for this analysis. The same statistical analysis procedure was followed for the notice rate using a Glimmix model (SAS 9.4). For all comparisons, significance was assessed at the 95% confidence level.

Results: For the fixation duration results, the mixed model showed that the fixed effects of colour (F(5, 545) = 5.73, p<0.0001) and order (F(5, 545) = 3.02, p = 0.01) were statistically significant. This means that colour significantly impacted the mean value of fixation duration as did the position of the capsule. The mixed model showed that the fixed effect of sequence (F(5, 105) = 1.06, p = 0.39) was not significant.

For the notice rate results the Glimmix model showed that the fixed effects of colour (F(5, 545) = 7.09, p < .0001) and order (F(5, 545) = 2.83, p = 0.02) were also statistically significant. Consistent with fixation duration it showed that the fixed effect of sequence (F(5, 105) = 1.06, p = 0.39) was not statistically significant.

The paired comparison results for fixation duration and notice rates between colours are summarised in Table 1. By Tukey HSD test, the fixation duration (M ± SD) (0.58±0.55) and % notice rate (76 ± 44.0) of the high contrast capsule were significantly greater than that of the blue capsule (0.28 ± 0.43, p<0.001 and 48 ± 50.0, p<0.001, respectively). The high contrast capsule fixation duration (0.58 ± 0.55) and % notice rate (76 ± 44.0) were significantly greater compared to the purple capsules (0.37 ± 0.42, p = 0.02 and 55 ± 49.7, p = 0.01). The fixation duration for the low contrast capsule (0.56 ± 0.46) was significantly greater than that of the blue capsule (0.28 ± 0.43 p = 0.001). The % notice rate of the low contrast capsule (80 ± 40.3) was significantly greater than the % notice rate of the blue (48 ± 50.0, p<0.0001), green (61 ± 50.6, p = 0.02) and purple capsule (55 ± 49.7, p = 0.001).

**Table 1:** Mean and standard deviation (M ± SD) values for toddler notice rate and fixation duration of six different coloured capsules, positioned in a circle, recorded by eye tracking within a high-resolution image.

	White (W)	Green (G)	Blue (B)	Purple (P)	High contrast (HC)	Low contrast (LC)
Fixation duration (sec) (M ± SD)	0.45±0.48	0.44±0.50	0.28±0.43	0.37±0.42	0.58±0.55	0.56±0.46
Notice rate (%) (M ± SD)	63±49.0	61±50.6	48±50.0	55±49.7	76±44.0	80±40.3

B≠HC means blue capsule significantly differs from HC capsule (p<0.05), P≠HC, B≠LC (p<0.05) and the % notice rates show G≠LC, B≠HC, B≠LC, P≠HC, and P≠LC (p<0.05).

For the mono-coloured capsules, the findings showed no statistically significant differences in the fixation duration that the toddlers spent looking at the mono-coloured capsules (p>0.07) or in the notice rates (p>0.06).

Confidence interval calculations for the paired comparisons of the six colours were completed. The range for the confidence intervals of fixation duration was between -0.49 to 0.35 and the range for the confidence intervals for the % notice rate was from -53 to 26. These ranges demonstrate relatively large variation in the fixation duration and notice rates between toddlers, compared to the minimum significant differences (0.19 seconds and 20%).

Findings: The fixation duration and notice rate were significantly greater for the high contrast capsule versus the blue and the purple. For the low contrast capsule a statistically significant difference in greater notice rates and fixation duration was shown versus the blue capsule. There were no statistically significant differences between the mono-coloured capsules.

### Study 2 –A more ecologically valid eye tracking study

Method: On the same television monitor, each toddler viewed six high resolution images of the six different colour capsules used in study 1. This time the capsules were shown in their open packaging (i.e. a box) along with capsules of the same colour placed in the kitchen cupboard set-up. Each of the six images was projected for four seconds, one after another in a pre-assigned sequence of six. This study measured visual attention using the fixation duration (seconds) and the % notice rate of the opening of the box displaying the capsules as the defined area of interest (see Figs 3 and 4).

Participants: The same toddlers participated in this study as in Study 1. However, the number of participants varied between the different images, as toddlers whose gaze was not focused on the screen were excluded from the analysis.

Stimulus materials: Six high resolution images of a kitchen cupboard set-up containing cleaning product facsimiles were used. Each cupboard image contained nine identical control household cleaning items (dishwasher salt, rinse aid, washing up liquid, gel floor cleaner, hand soap, floor wipes, surface wipes, dishcloth and washing-up sponges) and an open box displaying one of the six colours of capsules used in Study 1 (see Fig 3). The only variable between the images was the colour of the capsules displayed in the box (see Fig 4 as an example).

Procedure: In this study following calibration of the equipment, the six images were displayed on the screen, interspersed with a video of a circus cartoon of which the length was randomly varied from two to nine seconds. The ordering of the projected images was randomised using SAS9.4 software to ensure that each capsule colour shown was balanced and that it occurred at each possible position of the image sequence.

Statistical Analysis: A mixed model was used for statistical analysis of fixation duration. Within this model the colour, order and sequence of the six different images were taken as fixed effects; the participant as a random effect and fixation duration as a response variable. The order and sequence that the images were shown was randomised and balanced. JMP 13.1 was used for this analysis. The same statistical analysis procedure was followed for the notice rate using a Glimmix model (SAS 9.4). For all comparisons, significance was assessed at the 95% confidence level.

Results: For the fixation duration results, the mixed model yielded no significant effects for any of the fixed factors (order, F(5, 572) = 0.73, p = 0.60; sequence, F(5, 126) = 1.66, p = 0.15, or colour, F(5, 572) = 1.23, p = 0.29). Likewise, for the notice rate results, all of the fixed effects in the Glimmix model were not significant (order, F(5, 558) = 1.11, p = 0.35; sequence, F(5,126) = 0.87, p = 0.50; colour, F(5, 558) = 0.58, p = 0.72). The mean values and standard deviations for the fixation durations and notice rates between colours are summarised in Table 2. They show no statistically significant differences in either the fixation duration time spent looking at the different capsules, or the toddler notice rates.

**Table 2:** Mean and standard deviation (M ± SD) values for Toddler % notice rate and fixation durations of six different coloured capsules in their open box recorded by eye tracking within a high-resolution image of a kitchen cupboard.

	White	Green	Blue	Purple	High contrast	Low contrast
Toddlers (n)	118	121	109	113	118	121
Fixation duration (seconds) (M ± SD)	0.28±0.40	0.33±0.41	0.24±0.39	0.32±0.48	0.26±0.36	0.31±0.45
Notice rate (%) (M ± SD)	48±50.3	54±50.3	47±50.3	46±49.8	51±50.5	50±50.3

Confidence intervals for the paired comparison of the six different images were completed. The range for the confidence intervals of fixation duration was -0.22–0.23 and the range for the confidence intervals for the notice rate was -19%–22%. These ranges demonstrate relatively small variations in the fixation duration and notice rates between toddlers, compared to the minimum significant differences (0.19 seconds and 20%) and those found in Study 1.

Findings: No statistically significant differences were found in the toddlers’ fixation duration or notice rates for any of the different coloured capsules displayed in their open box in the cupboard set-up.

### Study 3 –Out of context behavioural observation study

Method: After completing study 4 and following a 20–30 minute break, Toddlers were sat on a stool at a toddler’s table for a controlled environment grasping choice task.

Participants: A total of 188 toddlers (mean age in months, M ± SD, 22.05 ± 6.47, 95 boys, 93 girls, R = 12–36) participated in this behavioural task. These toddlers included the 134 toddlers used earlier in studies 1 and 2, plus 54 additional toddlers who were recruited into study 3 and 4 to ensure the base size was robust.

Stimulus materials: This task involved six different arrangements (subsets) of three different coloured capsule replicas drawing from the six colours used in studies 1 and 2 (mono-coloured green, blue, white, purple, multi-coloured low and high contrast) using a randomisation pattern. Each time the capsule replicas were placed on set markers on a grey card attached to the table top in front of the seated toddler, to form a standardised line on a neutral background (see Fig 5).

Procedure: A MaxDiff procedure was used [14]. Each toddler was asked to “take the one capsule that you would most like to play with”, followed by a second choice. The first chosen capsule was defined as the most favoured, and the capsule not chosen at all from the three as the least. This task was repeated for the further five remaining subsets. The combined results from all six subsets were used to calculate the probability of choice for each of the six capsule colours.

Statistical Analysis: SSI web software version 9.1 [14, 15] was used to create the randomisation design and to calculate the probability of choice. A one-way ANOVA with the factor ‘Colour’ was performed using JMP13 to detect significant differences among the probabilities. For all comparisons, significances were assessed at the 95% confidence level.

Results: The ANOVA yielded a significant effect of the factor Colour, F(5, 1122) = 17.78, p<0.0001. Tukey HSD as post-hoc tests showed the pair comparison results in Table 3 for the probabilities of choosing each of the colours. The white, purple, and both contrast capsules were significantly preferred over the green or blue, all ps<0.01.

**Table 3:** Mean and standard deviation (M ± SD) values for probability of toddler choice of capsule in the out of context behavioural task.

Capsule colour	White (W)	Green (G)	Blue (B)	Purple (P)	High Contrast (HC)	Low Contrast (LC)
Probability of choice (M ± SD)	0.36±0.12	0.31±0.07	0.30±0.06	0.36±0.08	0.36±0.09	0.34±0.1

W≠G means the white capsule significantly differs from Green capsule (p<0.05). W≠B, G≠P, G≠HC, G≠LC, B≠P, B≠HC and B≠LC (p<0.05).

Findings: In an out-of-context set-up, the white, purple, and both contrast capsules were chosen with significantly higher probability than the green or blue capsules.

### Study 4: A more ecologically valid behavioural observation study

Method: This behavioural study assessed toddler grasping choices from the cupboard set-up used in study 2 for two of the six colour capsule replicas, by means of a between-subjects design. Green mono-colour and multi-coloured high contrast capsule replicas were used as the most differentiated in visual appearance.

Participants: The participant sample included 240 toddlers, 134 of which had also taken part in studies 1 and 2. A total of 118 toddlers (M ± SD, 21.81 ± 7.17; 57 boys, 61 girls) were assigned to the task using the green capsule, and 122 toddlers (M ± SD, 21.45 ± 6.96; 63 boys, 59 girls) to the task using the high contrast capsule.

Stimulus materials: The study employed two identical rooms (in terms of size, furniture and lighting) each with identical cupboard set-ups containing the nine cleaning product facsimiles as described in Study 2 (Fig 6). The cleaning products were chosen to represent typical items stored at home alongside laundry products. They were all of a size and weight that could be easily grasped by a toddler. All containers were cleaned and filled with water, milk or juice, coloured to mimic commercial products they were replacing (in the case of liquids) or inert solid items, e.g. small candles (in the case of solids). The items were arranged identically in each of the cupboards and the capsule replicas (either green mono-colour or multi-colour high contrast) were displayed in identical open packaging and placed in the same position amongst the nine items in the two cupboards. Half way through the study the colour of the capsules displayed in the box were switched to balance out any unforeseen effects of the room.

Procedure: Toddlers were allocated to one of the rooms and allowed up to 10 minutes to settle, so that they were at ease and able to play, during which time the open cupboard was concealed behind a screen. When the toddler had settled, the researcher moved closer to the cupboard and invited the toddler to “take one thing from the cupboard that you would most like to play with”. The position in front of the cupboard was the same for all toddlers and all items were reachable in principle. After the result was recorded, the toddler was asked to give the item to their caregiver, and the item was put to one side. The grasping choice task was repeated three times in total. The frequency with which either a single capsule or the complete box containing the capsules was chosen, was calculated for first, second and third choice. The sum of the three choices was then additionally calculated.

Statistical Analysis: The results of the two differently coloured capsules from the cupboards were compared using a Chi-square test (Fisher’s exact test) in JMP13 software. A Monte Carlo simulation was used to confirm the repeatability of the statistical results [16]. For all comparisons, significance was assessed at the 95% confidence level.

Results: The percentages of toddlers who chose the green capsules and the multi-coloured high contrast capsules among their first three chosen items from the cupboard are summarised in Table 4. Results are given for the percentage of toddlers grasping a single capsule, the complete box and for either of the two.

**Table 4:** Percentage of toddlers choosing the box displaying the capsules, the discrete capsules and the sum of the box or the capsule in the first three items chosen, separated by capsule colour.

Item	Box displaying capsule		Capsule		Box displaying capsule or individual capsule	
Toddlers (N)	111	118	111	118	111	118
Capsule colour	Green	High contrast	Green	High contrast	Green	High contrast
1^st^ chosen %	5%	6%	6%	10%	12%	16%
2^nd^ chosen %	4%	5%	6%	10%	10%	16%
3^rd^ chosen %	8%	6%	7%	4%	14%	10%
Chosen either 1^st^, 2^nd^ or 3^rd^ %	17%	17%	20%	25%	32%	33%

Table 5 shows the confidence interval of the differences between the probabilities of grasping the green capsule versus the multi-coloured high contrast capsule, the complete box displaying the green versus the multi-coloured high contrast capsules and the sum together. All confidence intervals include 0, and most of them had an upper or lower limit greater than 10%. This limit is relatively large, so a Monte Carlo simulation was run to explore the repeatability of the non-significant results.

**Table 5:** Confidence intervals for study 4.

Item	Box displaying capsule (green vs. high contrast)	Capsule (green vs. high contrast)	Box displaying capsule + capsule (green vs. high contrast)
Statistics	Confidence interval (%)	Confidence interval (%)	Confidence interval (%)
1^st^ chosen	(-7, 6)	(-11, 4)	(-13, 5)
2^nd^ chosen	(-7, 4)	(-11, 4)	(-14, 4)
3^rd^ chosen	(-5, 8)	(-4, 9)	(-5, 12)
% in Top 3 chosen	(-10, 10)	(-16, 6)	(-13, 12)

Table 6 shows the Chi-Square value, degree of freedom and p-value for each pair test. There were no statistically significant differences between the green and the multi-coloured high contrast results for the first three items chosen in terms of the percentage of toddlers choosing a single capsule, the box, or an individual capsule plus the box.

**Table 6:** Results of the Chi-square test performed in study 4.

Item	Box displaying capsule (green vs. high contrast)			Capsule (green vs. high contrast)			Box displaying capsule + capsule (green vs. high contrast)		
Statistics	Chi-square	Df	P value	Chi-square	Df	P value	Chi-square	df	P value
1^st^ chosen	0.03	1	0.86	1.14	1	0.29	0.92	1	0.34
2^nd^ chosen	0.29	1	0.59	1.09	1	0.30	1.43	1	0.23
3^rd^ chosen	0.19	1	0.67	0.55	1	0.46	0.71	1	0.40
% in Top 3 chosen	0.01	1	0.94	0.86	1	0.35	0.01	1	0.91

The Monte Carlo simulation was conducted with the observed probabilities from Table 4 using the largest difference between each pair of probabilities (≤6%). The results shown in Table 7 are the probabilities that the results can be repeated under the same test conditions. The results show overall that there was over a 70% power to repeat the non-significant results.

**Table 7:** Monte carlo results predicting repeatability within a 6% range.

Item	Box displaying capsule	Capsule	Box displaying capsule or capsule
Toddlers (N)	111 vs. 118	111 vs. 118	111 vs. 118
Capsule colour	Green vs. high Contrast	Green vs. high contrast	Green vs. high contrast
1^st^ choice	95%	73%	66%
2^nd^ chosen	96%	73%	46%
3^rd^ chosen	87%	86%	68%
% in Top 3 chosen	74%	55%	64%

Findings: No statistically significant differences were found in the percentage of toddlers grasping the green or multi coloured high contrast capsules from the cupboard.

## Discussion

This research set out to formally explore if differences in colours and contrasting colour designs used in mono and multi-coloured liquid laundry capsules result in different levels of toddler attractiveness to better understand market accidentology data which, contrary to opinion expressed in the media [1, 2], show no relation between incident frequency rate and capsule colour [13]. This work was completed to contribute to a broader study designed to help uncover the real nature as to what drives toddler interaction with laundry capsules that may lead to poisoning incidents in the home to help develop counter-measures. The research consisted of four independent studies that examined how toddlers responded to different coloured capsules using a variety of methods.

Eye tracking was used to measure variations in visual attention and notice rate of toddler’s when looking at different coloured capsules to determine if any colours were particularly preferred. Behaviour observation tasks then measured the degree in which different coloured capsules drove the act of grasping, which examined if particular colours also manifested in different levels of a toddler’s desire to physically interact with them (which could lead to a potential poisoning incident). These tasks were completed in both a neutral out of context set-up and in a more ecologically valid setting to help mimic a realistic home laundry environment.

The studies completed in the out of context set-up, showed that the colour of the capsule had a significant impact on both the visual attention and the grasping choice, but that visual attention may not necessarily be predictive of the act of grasping. These results were at odds from those in a more ecologically valid setting which demonstrated both that the colour of the capsule did not have a significant impact, and that the visual attention may be a good predictor of grasping. Importantly, the results from the more ecologically valid setting were consistent with market accidentology data which also show no impact of colour to incident rates.

In the out of context eye tracking study the capsules with the multi-coloured high and low contrast designs were noticed by a higher percentage of toddlers and elicited longer looking times versus some of the mono-colour capsules (high contrast–blue and purple, low contrast–blue). No differences were found between the mono-coloured capsules, i.e. green, blue, purple and white. In the out of context behavioural study examining grasping choice, the multi-coloured high and low contrast designs were chosen with a higher probability than the mono-coloured blue or green. The mono-coloured white and purple were also chosen with higher probabilities than the others, along with the high and low contrast designs. These results suggest that whereas colour may play a role in visual attention and grasping choice, results achieved in visual attention experiments may not necessarily be predictive of grasping choice.

Taylor et al. highlight the increased noticing by toddlers may not necessarily indicate liking, but may instead reflect other factors, such as complexity, salience or novelty–in other words, their increased noticing may have more to do with objects being different or unusual, and so ‘catching their eye’ [17]. This may explain why in the out of context study when attention is focused on the capsules without the ‘distraction’ of the ecological set-up (which involves more parameters), the multi-coloured contrast capsules received higher levels of visual attention and notice rates than the mono-coloured capsules. In the grasping choice task, the white and purple mono-coloured capsules were also chosen more than green or blue. This demonstrates differences in the single act of noticing and its relationship to the more complex behaviour of reaching out and grasping. It is the latter which is most important to understand in order to prevent poisoning incidents.

In the more ecologically valid eye tracking study no statistically significant differences between the six different coloured capsules were found in the fixation duration or % notice rates of the toddlers. Also, in the more ecologically valid grasping choice task no differences in the probability of choice were observed between the two capsules studied, that is the mono-coloured green and multi-coloured high contrast capsules. We are confident that these results reflect the real situation given that the mean differences between the different coloured capsules found in the studies are small, confidence intervals are narrow and that the results were achieved within the context of large sample sizes. Additionally within the grasping choice exercise where a Monte Carlo simulation was completed, results confirmed high repeatability.

The more ecologically valid study findings were aligned with real-world poisoning incident numbers, where the trends in unintentional interaction by toddlers leading to reported incidents, show no consistent differences between the most prevalent colours (blue, green, white, purple and multi-colour contrast capsules [13]. The results of these studies on toddler visual attention and preference for differently coloured capsules, conducted in both out of context and in more ecologically valid settings, suggest that the out of context findings may not be a reliable indicator of real-world behaviour, and the interpretation of these results to predict any impact on the accidentology rate should be viewed with caution.

There are several limitations to this study. Firstly, although the studies offer more ecological validity than those performed in a laboratory setting, they were not conducted within an at-home setting with all its contextual features. The cupboard set up in the study may be considered open to interpretation as everyone will organise their cleaning cupboard differently, and it could argued that participants would not necessarily feel as comfortable or be as natural “playing” in this simulated situation as they would be in their own home. Each participant was asked to choose an object from the cupboard or a replica liquid laundry capsule from a line-up as part of the studies, however, poisoning incidents in the home are more likely to take place whilst the toddler is without adult supervision. Another limitation is that participants during the out of context eye tracking study (study 3) were looking at 2D images of liquid laundry capsules which may lose certain characteristics of the actual 3D object, such as distinctive texture, lustre, etc. that could be interesting to a toddler.

## Conclusion

The out of context findings of a statistical preference for multi-coloured capsules in Study 1, and in the case of Study 3, of a statistical preference for mono-coloured white and purple as well as multi-colours over green and blue, showed that colour may have an impact on the level to which toddlers may be attracted to capsules but that visual attention may not necessarily correlate with the act of grasping. These colour preference results are not aligned with real-world data. In comparison, no statistical differences in levels of attractiveness when looking at mono-coloured or multi-coloured capsules were found when investigated within a more ecologically valid setting (Studies 2 and 4). Within this context visual attention was predictive of grasping and importantly matched in-market, real-world data. Overall, the data from the more ecologically designed studies do not support a link between the colour of capsules and differences that colours may elicit in either visual attention or grasping–with its associated risk of poisoning incidents. The findings suggest that caution should be exercised in extrapolating outcomes from research conducted in an out of context setting to predict the risk of poisoning incidents in the home, and recommends a more ecological study design with a valid real-world setting to do this.

This work was completed to contribute to a broader study designed to help uncover what drives toddler interaction with laundry capsules that may lead to poisoning incidents in the home to help develop effective counter-measures. Findings support the importance of child-safe packaging and parental awareness as strategies to prevent unintentional interactions (which have led to a statistical reduction in incidents in the market place), rather than changes in capsule colour design.

## Supporting information

S1 Fig Study 1 raw data. (XLSX)

S2 Fig Study 2 raw data. (XLSX)

S3 Fig Study 3 raw data. (XLSX)

S4 Fig Study 4 raw data. (XLSX)

S5 Fig Supporting statistical data. Supporting statistical data from all studies.
(XLSX)
